# A high rate of falls and traumatic fractures occurs after extensor mechanism reconstruction: a cohort study including both allograft and synthetic mesh grafts

**DOI:** 10.1186/s42836-026-00393-8

**Published:** 2026-06-01

**Authors:** Sebastian Braun, Isiah Selkridge, Allina Nocon, Peter K. Sculco

**Affiliations:** 1 Stavros Niarchos Foundation Complex Joint Reconstruction Center, Hospital for Special Surgery, New York, NY 10021 USA; 2 Center for Musculoskeletal Surgery, Member of Freie, Charité Universitätsmedizin Berlin, Universität Berlin and Humboldt University Berlin, 10117 Berlin, Germany

**Keywords:** Extensor mechanism reconstruction, Total knee arthroplasty, Revision total knee arthroplasty, Total knee arthroplasty complications

## Abstract

**Background:**

Extensor mechanism disruption following total knee arthroplasty (TKA) significantly impairs knee function and quality of life. Extensor mechanism reconstruction (EMR) is effective but carries risks, including post-operative falls due to persistent quadriceps weakness and extensor lag. We sought out to answer the following four questions: What is the incidence of traumatic events following EMR after TKA using different grafting and reconstruction techniques? Are there significant differences in the rate of traumatic events based on the type of graft used (allograft vs. mesh graft) in EMR? What is the association between post-operative extensor lag and the occurrence of traumatic events in patients who have undergone EMR? Does the use of assistive devices influence the occurrence of traumatic events post-EMR?

**Methods:**

This retrospective cohort study at an academic center included 41 patients (mean age: 67.8 ± 10.1 years; 61% female) who underwent EMR post-TKA at a single academic center. Reconstructions included allograft (*n* = 25) and synthetic mesh grafts (*n* = 16). Patient demographics, ASA Score, type of EMR, and post-operative extensor lag were documented. The primary outcome was the occurrence of traumatic events post-EMR. Statistical analysis involved Fisher’s exact test, with *p* < 0.05 considered significant.

**Results:**

Of the 41 patients, 16 (39%) experienced post-operative falls leading to traumatic injuries. No significant differences were found in traumatic event rates between the allograft (36%) and mesh graft (43.75%) groups. The mean extensor lag was 7° ± 14°, with no statistically significant association observed between the degree of extensor lag and traumatic events. The use of assistive devices did not significantly influence the occurrence of traumatic events.

**Conclusion:**

More than one-third of patients experienced traumatic events following EMR after TKA, highlighting the need for comprehensive post-operative management and patient counseling. The study found no statistically significant association between graft type, extensor lag, or use of assisted devices and the occurrence of these events. Further research is required to understand the risk factors and improve patient outcomes in this clinically challenging domain.

## Background

Extensor mechanism disruption (EMD) after total knee arthroplasty (TKA) is a devastating complication that represents a significant clinical challenge with long-term consequences that substantially impair knee function, ability to ambulate unassisted, and overall quality of life [1, 2]. Extensor mechanism (EM) complications include patellar fractures, patellar tendon and quadriceps tendon ruptures, and the surgical reconstruction of these injuries, while often effective in restoring knee function, is not without risk [3]. Indeed, post-operative complications and reoperations are common, as previous research highlights [3–6]. One post-operative complication associated with extensor mechanism reconstruction (EMR) is the increased risk of post-operative falls, likely related to persistent quadriceps muscle weakness and extensor lag. Moreover, the increased likelihood of falls leading to significant injury after an EMR has not been previously reported in the orthopaedic literature.

While it is known that persistent extensor lag increases the risk of knee buckling and falls, we do not fully know the frequency or effect of falls on patient outcomes. Such research could guide clinicians in identifying high-risk patients, thereby tailoring perioperative care and counseling to mitigate the risks of adverse events and improve overall patient outcomes. The goal of this study was to highlight the increased risk of mechanical falls and subsequent traumatic events in patients who have undergone an EMR after TKA. We sought out to answer the following four questions:

1. What is the incidence of traumatic events following EMR after TKA using different grafting and reconstruction techniques?
2. Are there significant differences in the rate of traumatic events based on the type of graft used (allograft vs. mesh graft) in EMR?
3. What is the association between post-operative extensor lag and the occurrence of traumatic events in patients who have undergone EMR?
4. Does the use of assistive devices (like canes, walkers, or wheelchairs) influence the occurrence of traumatic events post-EMR?

## Material and methods

### Study design and setting

The study received approval from our institution’s review board (study no. 2022-2137). We conducted a retrospective cohort study to assess the incidence of traumatic events following EMR of the knee following TKA using different grafting and reconstruction techniques. The study was conducted at an academic primary care hospital; the hospital database was retrospectively reviewed for cases from January 2017 to November 2022. Patient demographics were recorded, including age, body mass index (BMI), and sex. ASA (American Society of Anesthesiologists) Score, type of surgery (primary EM surgery vs. revision EM surgery following TKA), type of EMR (allograft vs. mesh graft), and post-operative extensor lag recorded at 6 weeks to 12 months post-operative were documented. The study was approved by the Institutional Review Board, and all procedures adhered to the Declaration of Helsinki.

### Patient cohort

Patients who underwent EMR for chronic EMD after TKA were identified from the hospital database using relevant procedure codes. Chronic EMDs were defined as injuries occurring more than six weeks prior to surgical management. Both complete and partial chronic EMD were included. Chronic EMD was diagnosed clinically by the presence of extensor lag and confirmed by ultrasound and/or magnetic resonance imaging (MRI). Partial EMDs were included if they were associated with severe symptoms and functional disability, including significant impairment in active knee extension and ambulation, and where surgical reconstruction was deemed necessary. In the absence of a universally accepted cutoff for post-operative extensor lag, a threshold of > 10° was pragmatically applied to distinguish between physiological and clinically relevant extensor mechanism dysfunction and to form the basis for the stratified analysis presented in Table 3 [7, 8]. In contrast, EM failure was defined as the inability to achieve active knee extension associated with clinically significant extensor lag and functional limitation; consistent with prior reports, an extensor lag exceeding 30° was considered a marker of treatment failure [29, 30]. Inclusion criteria were adult patients (≥ 18 years) who had a prior TKA and suffered an EM injury and had a minimum follow-up of one year post-operatively. Exclusion criteria included patients with systemic inflammatory diseases or those who did not complete the required follow-up. Potential sources of bias, such as information bias from retrospective data collection and potential selection bias, were acknowledged. Efforts to minimize bias included rigorous data validation through cross-referencing and validating patient reports with clinical notes. We identified 41 patients who met the inclusion criteria. For all patients, clinical notes and radiological reports were reviewed, and patients were contacted by phone if follow-up information was insufficient.

### Surgical techniques

Allograft [9]: The fresh-frozen allograft was procured from a tissue bank, screened for diseases, and sized to the recipient’s anatomy. The graft was thawed, prepared, and anchored to the native bone using screws or suture anchors; no autografts were utilized in this cohort. For the mesh graft [8]: A synthetic mesh was sized and cut to the defect’s dimensions. The mesh was either cemented into a tibial trough or inserted into the tibial canal, if a concomitant component revision was performed, and then sutured into the quadricep in a pants-vest fashion and secured under tension with non-absorbable sutures. The allograft and mesh graft were tensioned with the leg in full extension and sutured to host EM tissue according to previously published surgical techniques. The indication for EMR was based on the presence of a functionally deficient EM with the inability to extend the knee actively, extensor lag, and associated functional impairment. The choice between allograft and synthetic mesh reconstruction was made at the discretion of the treating surgeon based on intra-operative findings, tissue quality, and patient-specific factors. In general, allograft reconstruction was preferred in cases with substantial tissue loss or poor native tendon quality where biological augmentation was deemed beneficial. Synthetic mesh reconstruction was more commonly utilized in cases where host tissue was insufficient for primary repair but where a synthetic augmentation was considered adequate to restore EM continuity. No strict standardized protocol for graft selection was applied.

### Outcome measures

The primary outcome was the occurrence of traumatic events after EMR. For this study, a traumatic event was defined as any unplanned mechanical fall or external injury occurring after EMR that resulted in a musculoskeletal injury or other injuries requiring medical evaluation or a documented change in post-operative management and were captured over the entire postoperative follow-up period. In addition, the clinical outcome after patient falls was recorded, and whether the fall was associated with fracture or other injury that required surgical management. Data collection included patient demographics, type of graft used, post-operative complications, and the occurrence of traumatic events and further surgery. The use of an assisted devices, both prior to and after falling, was recorded as either a cane, walker, or wheelchair. This information was extracted from the hospital’s electronic medical record system and, when needed, through telephonic interviews with patients.

### Statistical analysis

Descriptive statistics, including frequencies, percentages, and cumulative percentages, were used to summarize the data. A Fisher’s exact test was employed to determine the association between graft type and the occurrence of traumatic events. Statistical analysis was performed with SAS version 9.3 (Cary, NC, USA) with a level of significance assumed at *p* < 0.05.

## Results

### What is the overall incidence of traumatic events following EMR after TKA?

Forty-one participants were included in the study. Demographic analysis showed two distinct graft groups: Allograft (*n* = 25) and Mesh (*n* = 16), Table 1. The average follow-up time was consistent across all groups at 3.7 (+/− 1.73) years. The study focused on traumatic events post-surgery. Of the total cohort undergoing extensor mechanism reconstruction, 16 patients (39%) experienced a post-operative fall leading to a traumatic injury. Each traumatic event occurred in a unique patient, with no patients experiencing multiple events during the follow-up period.

**Table 1:** Baseline patient demographics, surgical characteristics, and overall incidence of traumatic events following extensor mechanism reconstruction

**Parameter**	**Mean**	**SD**	**Range**
Age	67.76	10.05	47.70–87.80
BMI	33.78	6.81	20.20–47.40
ASA	2.50	0.50	1–4
		Male	Female
Sex	N = 41	16 (39.02 %)	25 (60.98 %)
		Graft	Mesh
Type of EMR procedure	N = 41	25 (60.98 %)	16 (39.02 %)
		Primary	Revision
Type of EM surgery following TKA	N = 41	22 (53.66 %)	19 (46.34 %)
Traumatic Events		Graft	Mesh
Yes	N = 16	9 (36.00 %)	7 (43.75 %)
No	N = 25	16 (64 %)	9 (56.25 %)

SD – standard deviation, BMI – body mass index, ASA – American Society of Anesthesiologists, EMR – extensor mechanism reconstruction, EM – extensor mechanism, TKA – total knee arthroplasty

### Are there significant differences in the rate of traumatic events based on the type of graft used (allograft vs. mesh graft) in EMR?

In the allograft group (*n* = 25), 16 (64%) participants reported no events, while 9 (36%) reported traumatic events. Regarding the Mesh group (*n* = 16), 9 (56.25%) participants reported no event, with 7 (43.75%) suffered a fall. Subgroup analysis, stratified by graft type, did not indicate any significant differences in traumatic event rate within each subgroup (*p* = 0.6197). Table 2 shows further exploration of specific traumatic events and the distribution of interventions following these traumatic events.

**Table 2:** Types of traumatic events and subsequent management following extensor mechanism reconstruction

		**Frequency**	**Percent**
Traumatic event outcome	No radiographic injury	6	37.50
	Ankle fracture	2	12.50
	Extensor mechanism failure*	3	18.75
	Periprosthetic femur fracture	2	12.50
	Fibular spiral fracture	1	6.25
	Shoulder Dislocation	1	6.25
	Knee avulsion fracture, facial contusion, Ankle fracture	1	6.25
Traumatic event intervention	No intervention	9	56.25
	Cast immobilization	1	6.25
	Extensor mechanism reconstruction	3	18.75
	Open reduction internal fixation	3	18.75

Percentages are calculated using the total number of traumatic events (*n* = 16) as the denominator

^*^Extensor mechanism failure cases reported in this table represent traumatic re-ruptures occurring after a documented fall

### What is the association between post-operative extensor lag and the occurrence of traumatic events in patients who have undergone EMR?

Forty-one patients experienced a mean extensor lag of 7° ± 14°. Eight patients (19%) exhibited a post-operative extensor lag of > 10° from 6 weeks post-operative to one year, and 33 patients (81%) had an extensor lag < 10°, see Table 3. Within the cohort of > 10°, 4 patients exhibited an extensor lag > 30°; these patients experienced no fall or traumatic event. When comparing the rates of traumatic events between patients with and without a post-operative extensor lag, the data suggest no significant difference (10° cut off, *p* = 0.114). This suggests that the degree of extensor lag may not be a reliable predictor of falls and risk for traumatic events post-EMR.

**Table 3:** Association between postoperative extensor lag and occurrence of traumatic events

Extensor lag	Traumatic events		Total	*p*-value
	**Yes**	**No**		
> 10° (6 weeks until one year postoperative)	1 (2%)	7 (17%)	8 (19%)	0.114
< 10° (6 weeks until one year postoperative)	15 (37%)	18 (44%)	33 (81%)	
Total	16 (39%)	25 (61%)	41 (100%)	

### Does the use of assistive devices influence the occurrence of traumatic events post-EMR?

Table 4 summarizes the distribution of traumatic events among patients using different assistive device categories (none, cane, walker, wheelchair). Stratified into two groups (1. none and/or cane, and 2. all other), there was no statistical difference for traumatic events based on use of an assistive device, *p* = 0.1242 (Table 5). The analysis found no significant difference in traumatic events based on the use of devices (*p* = 0.4526) or when categorized into none and/or cane and all other devices (*p* = 0.1242).

**Table 4:** Distribution of traumatic events according to the type of assistive device used

Assisted device	Traumatic events		Total	*p*-value
	**Yes**	**No**		
None	5 (12.20%)	12 (29.27%)	17 (41.46%)	0.4526
Cane	2 (4.88%)	5 (12.20%)	7 (17.07%)	
Walker	6 (14.63%)	4 (9.76%)	10 (24.39%)	
Wheelchair	3 (7.32%)	4 (9.76%)	7 (17.07%)	
Total	16 (39.02%)	25 (60.98%)	41 (100%)

**Table 5:** Association between grouped assistive device use and occurrence of traumatic events

Assisted device	Traumatic events		Total	*p*-value
	**Yes**	**No**		
None + Cane	7 (17.07%)	17 (41.46%)	24 (58.54%)	0.1242
All other	9 (21.95%)	8 (19.51%)	17 (41.46%)	
Total	16 (39.02%)	25 (60.98%)	41 (100%)	

## Discussion

The current study investigated traumatic events following EMR of the knee, a topic that remains insufficiently explored in the orthopaedic literature, and should be interpreted as a descriptive cohort analysis. In our cohort of 41 patients, more than one-third experienced at least one traumatic event postoperatively, and one in seven sustained an injury that required operative intervention. The severity of trauma sustained by the study population underscores the clinical relevance of understanding functional outcomes following EMR and highlights that steps must be taken to prevent these adverse outcomes.

Risk factors for falls after primary TKA and total hip arthroplasty (THA) include certain medications and polypharmacy, mental health conditions, alcohol abuse, living circumstances, history of falls, and gender [10, 11]. However, these insights have not been explored in patients after revision TKA or those who have had an EMD after TKA and subsequent reconstruction. The EM serves the crucial function of stabilizing the knee in extension and preventing buckling during the heel strike phase of gait; however, empirical evidence regarding fall risk in this specific surgical cohort is scarce.

Post-operative complications following extensor mechanism repair or reconstruction include infection, structural failure, and persistent extensor lag. Patient-related factors such as age, sex, smoking status, diabetes mellitus, and obesity have been associated with an increased risk of adverse outcomes [12]. Despite advances in surgical techniques, both allograft and synthetic reconstructions demonstrate only moderate effectiveness, with considerable rates of complications and reoperations reported in the literature [3].

Allograft-based approaches for EMR, including Achilles tendon allograft (ATA) and whole extensor mechanism allograft (EMA), have shown variable and often inconsistent outcomes [4]. Synthetic mesh reconstruction, such as Marlex mesh, represents an established alternative, with a reported mean post-operative extensor lag of approximately 9° in large institutional series [8]. Persistent extensor lag remains a common limitation across both techniques and may negatively impact functional outcomes and contribute to an increased risk of falls [4].

In the present study, no statistically significant association was found between graft type (allograft vs. mesh) and the occurrence of traumatic events (*p* = 0.6). This finding is consistent with prior studies demonstrating no significant differences in clinical outcomes, survivorship, or complication rates between allograft and mesh reconstructions [25]. Accordingly, graft selection may be guided primarily by patient-specific factors, tissue quality, and surgeon preference rather than expectations of differential risk for post-operative traumatic events; however, these findings should be interpreted with caution, given the limited sample size. Both allograft and synthetic reconstructions have been shown to provide more reliable outcomes compared to primary repair in the setting of TKA, where tissue quality is often compromised [3, 4, 13, 14].

While allografts may offer advantages in restoring extensor mechanism function and improving host tissue integration, their availability remains limited in certain healthcare systems. In such settings, synthetic mesh reconstruction represents a practical and widely accessible alternative. In line with existing literature and our findings, comparable rates of traumatic events between graft types support the use of synthetic grafts without an apparent increase in post-operative risk [25].

For those facing chronic EMD post-TKA, allograft or synthetic graft reconstructions are often recommended as the most viable treatment options [16–19]. Overall, EMR in the setting of TKA remains a challenging clinical problem, associated with substantial morbidity and only moderate success rates [3, 9, 20–22]. Even in larger series, success rates remain limited, and traditional outcome measures such as in situ survival may not fully capture functional deficits and complication burden [3][23, 24]. Although ATA is often considered a reference standard for chronic reconstructions [15], no clear consensus exists regarding the optimal surgical technique [9, 15, 26–28]. Consequently, treatment strategies should be individualized based on patient characteristics, tissue quality, and surgical context [9, 15, 26–28].

In the review conducted by Balato et al. [4], it was reported that allograft ruptures due to traumatic injuries occurred in 3% (range 0–25%) of patients in the EMA group and 4% (range 0–10%) in the ATA group. In contrast, our study had only one instance of a traumatic quadriceps tendon rupture (2%). The traumatic events sustained by the patients in our study cohort included ankle fractures and peri-prosthetic femur fractures treated with open reduction and internal fixation (ORIF), knee avulsion fractures, and shoulder dislocations. The occurrence of a wide spectrum of traumatic events suggests that complications are not limited to the surgical site but may affect the patient’s overall physical stability. This further emphasizes the need for a comprehensive, multidisciplinary approach to post-operative care and rehabilitation to minimize the risks of falls and other traumatic events. Remarkably, 9 out of 16 patients (56.25%) in our study who experienced a fall or other traumatic event did not sustain any injuries or damage.

Extensor lag greater than 30° following EMR is widely regarded as indicative of treatment failure [29, 30], whereas smaller degrees of lag (e.g., > 10°) reflect clinically relevant impairment without necessarily constituting failure. Notably, four patients in our cohort exhibited this degree of extensor lag and subsequently experienced no falls. This intriguing observation raises the possibility that an increasingly compromised functional status, as indicated by more severe extensor lag, may serve as a protective factor against falls. It is conceivable that patients with substantial extensor lag may exhibit higher levels of dependency and reduced mobility, thereby adopting more cautious postures and placing themselves in less precarious situations compared to those with near-normal knee extension, who might tend to be more active. Patients who exhibited greater extensor lag (8 patients with a 10° cutoff) demonstrated a reduced inclination to experience falls. While our findings suggest a higher rate of traumatic events in patients with lower postoperative extensor lag (< 10°) compared with those with greater extensor lag (> 10°), this difference did not reach statistical significance (*p* = 0.114) and should be interpreted cautiously given the small sample size. Conversely, when examining the impact of assistive devices on traumatic events, our analysis did not find the use of an assistive device to be protective. Specifically, our findings suggest that the use of assistive devices may not have a substantial influence on the occurrence of traumatic events. However, it’s crucial to acknowledge the limitations inherent in our study, particularly the relatively small sample sizes within specific device categories, such as canes, walkers, and wheelchairs. Consequently, further research with expanded sample sizes is warranted to draw more definitive conclusions regarding the relationship between the use of these devices and the incidence of traumatic events.

This study has several limitations, including its retrospective design, which opens the possibility of information, recall, and selection bias. Efforts were made to minimize known sources, including rigorous data validation and direct patient contact; some biases likely persisted. Furthermore, extensor lag was assessed only within the early postoperative period (6 weeks to 12 months) and may therefore not accurately reflect extensor mechanism function at the time of later traumatic events occurring beyond the first postoperative year. The study sample was relatively small, and the post-operative follow-up varied, affecting the robustness of our conclusions. Lastly, as the study was conducted in a single academic primary care hospital, the generalizability of these results may be limited.

The relatively high rate of post-operative traumatic events observed in this study is likely multifactorial. Patients undergoing EMR often exhibit persistent quadriceps weakness, extensor lag, and impaired neuromuscular control, contributing to instability and increased fall risk. Altered gait patterns and reduced proprioception may further predispose patients to these events. Management depended on injury severity, with most cases treated conservatively and a subset requiring surgical intervention. The relationship between extensor lag and traumatic events remains complex. While extensor lag may predispose to falls, traumatic events may also impair extensor mechanism function. In this study, no statistically significant association was observed, suggesting that additional factors contribute to fall risk in this population. Notably, patients with better apparent functional recovery, including reduced extensor lag and less reliance on assistive devices, may paradoxically be at increased risk of falls, highlighting the need for a nuanced post-operative strategy. These findings emphasize the importance of rigorous post-operative monitoring and patient counseling following EMR in the setting of TKA. Targeted preventive strategies, such as balance training, optimization of comorbidities, and medication review, may help mitigate fall risk. Clinicians should consider incorporating structured fall prevention programs into post-operative care. Given the high incidence of traumatic events observed, further prospective studies are warranted to better define risk factors and improve patient outcomes.

## Conclusion

This study highlights the significant incidence of falls causing operative injuries following EMR in the setting of TKA. A comprehensive multidisciplinary approach is required in the early post-operative phase of care to optimize functional outcomes and minimize the risk of traumatic events. Further research is urgently needed to better understand the complex interplay of risk factors that contribute to these adverse outcomes, with the ultimate aim of improving patient care within this clinically challenging domain.

## Data Availability

The dataset used and/or analysed during the current study are available from the corresponding author on reasonable request.

## Abbreviations

EMD: extensor mechanism disruption
EMR: extensor mechanism reconstruction
EM: extensor mechanism
TKA: total knee arthroplasty
ASA: American Society of Anesthesiologists
BMI: body mass index
THA: total hip arthroplasty
